# Dietary arginine modulates egg production and mTOR signalling pathway gene expression in adult Japanese quail (*Coturnix japonica*)

**DOI:** 10.1016/j.vas.2026.100567

**Published:** 2026-01-05

**Authors:** Mequanint Gashew, Gebrehaweria K. Reda, Fadella Nur Almira, Eman Moustafa Abdelbary, Gabriella Gulyás, Renáta Knop, Brigitta Csernus, Csaba Szabó, Ádám Z. Lendvai, Levente Czeglédi

**Affiliations:** a Department of Animal Science, Institute of Animal Science, Biotechnology and Natural Conservation, Faculty of Agricultural and Food Sciences and Environmental Management, University of Debrecen, 4032 Debrecen, Hungary; b Doctoral School of Animal Science, University of Debrecen, 4032 Debrecen, Hungary; c Department of Animal Science, Agriculture and Environmental Science College, Debre Tabor University, 272 Debre Tabor, Ethiopia; d Department of Evolutionary Zoology and Human Biology, University of Debrecen, 4032 Debrecen, Hungary; e Department of Animal Nutrition and Physiology, Faculty of Agriculture and Food Sciences and Environmental Management, University of Debrecen, Debrecen 4032, Hungary

**Keywords:** Japanese quail, Arginine, mTOR, Gene expression

## Abstract

Arginine, an essential amino acid in birds, plays a central role in growth and reproduction. Regulation of the mechanistic target of rapamycin (mTOR) signalling pathway is among its key functions. This study examined how dietary arginine affects reproductive performance, body mass, and hepatic gene expression in adult female Japanese quail (*Coturnix japonica*). A total of 81 quails were randomly assigned to one of the three dietary treatments for 14 days: a diet containing 1.26% arginine (control), 0.945% arginine (restricted), and 1.575% arginine (supplemented). Body mass, hen-day egg production (HDEP), and egg mass were recorded, and liver samples were analysed using qPCR for mTOR signalling pathway-related genes. Arginine supplementation significantly increased HDEP, whereas neither restriction nor supplementation altered body mass or egg mass. Arginine restriction downregulated the expression of *MTOR, IGF1, GHR, FASN, ATG5, ATG9A, SOD1*, and *SOD2* genes. In contrast, supplementation upregulated *ATG5* but downregulated *SOD2*. Network analysis revealed treatment-dependent shifts: under restriction, *SOD2* functioned as a key bridge despite reduced expression, whereas under supplementation, centrality shifted to *GHR* and *FASN*, indicating enhanced growth and lipogenic activity. Overall, dietary arginine reshaped the molecular structure of the mTOR pathway without affecting somatic maintenance, instead favouring reproductive output. These findings suggest that dietary arginine regulates egg production and gene expression in the mTOR signalling pathway in adult Japanese quail.

## Highlights

• Body and egg mass were unaffected by 25% arginine restriction or supplementation.
• Arginine supplementation increased hen-day egg production percentage.
• Arginine restriction downregulated *MTOR, GH, IGF1*, and lipid synthesis genes.
• *GH* and *FASN* appeared as central nodes under arginine supplementation.

## 1 Introduction

Nutrition influences organismal development, growth, reproduction, and health by modulating molecular and phenotypic plasticity. Imbalances in nutrient availability, whether excess or deficiency, can disrupt physiological and metabolic homeostasis. Therefore, organisms adjust gene expression to adapt their energy metabolism to current conditions through molecular and physiological processes (Nourmohammad et al., 2017). Among essential nutrients, amino acids play a pivotal role in regulating the mechanistic Target of Rapamycin (mTOR) signalling pathway, which orchestrates key processes including growth, autophagy, and antioxidant responses (Takahara et al., 2020). Because of its central role in nutrient sensing, increasing attention has been given to how this pathway integrates amino acid availability with cellular metabolism. The mechanistic target of rapamycin (mTOR) pathway functions through two distinct complexes: mTOR complex 1 (mTORC1) and mTOR complex 2 (mTORC2). Of these, mTORC1 is especially sensitive to nutrient status, responding to amino acids, cellular energy, and stress signals to regulate protein synthesis, lipid metabolism, and autophagy (Avruch et al., 2009; Takahara et al., 2020). Notably, mTORC1 activation occurs through mechanisms distinct from insulin signalling (Nobukuni et al., 2005), highlighting its unique role in nutrient sensing. mTOR, a highly conserved serine/threonine kinase, is modulated by essential amino acids like leucine, methionine, glutamine, asparagine and arginine (Meng et al., 2020; Ndunguru et al., 2024a; Ndunguru et al., 2024b; Takahara et al., 2020). Studies across taxa demonstrate that arginine availability modulates mTOR activity and downstream gene expression dose-dependently, with both deficiency and excess eliciting distinct physiological responses (Gao et al., 2018; Kim et al., 2008; Wang et al., 2024; Zhou et al., 2024).

Cellular availability of amino acids, particularly mTOR-activating ones like arginine, triggers mTOR-mediated phosphorylation of downstream effectors (e.g., EIF4EBP1), thereby promoting protein synthesis while suppressing autophagy-related genes (Ndunguru et al., 2024b; Reda et al., 2024a; Yang et al., 2022). Conversely, amino acid restriction inhibits MTOR, activating protein recycling pathways, including autophagy, that help sustain cells under stress (Livneh et al., 2023; Wang et al., 2017). These responses are further modulated by age and physiological state, highlighting the complexity of nutrient-gene interactions.

While mTOR regulation is well-characterised in mammals, birds present a unique paradigm due to their lack of endogenous arginine synthesis, an evolutionary consequence of their incomplete urea cycle (Fernandes & Murakami, 2010; Silva et al., 2012). This constraint necessitates a higher dietary arginine intake compared to mammals (de Lima et al., 2022), yet its effects on adult birds remain poorly understood (Ball et al., 2007). Birds are underutilised models for studying nutrient-sensing evolution, and investigating the role of mTOR in this lineage is particularly important because of their unique physiology (Reda et al., 2024b). Their reliance on uric acid for nitrogen excretion, exceptionally high metabolic rates, and rapid developmental transitions provide a distinct framework to explore how mTOR signalling adapts to ecological constraints. Japanese quail (*Coturnix japonica*) is particularly valuable for such studies due to its small size, short generation time, and sensitivity to dietary manipulation (Caetano-Anolles et al., 2015; Minvielle, 2004; Vitorino Carvalho et al., 2019). Importantly, most avian research on arginine has focused on chicks (Cuca & Jensen, 1990; D’Amato & Humphrey, 2010), leaving a critical gap in understanding how adults, which face trade-offs between reproduction and self-maintenance, regulate the mTOR pathway.

To address this knowledge gap, we examined the physiological and molecular responses to dietary arginine variation in adult female quail. Consequently, we selected a panel of genes representing the major functional cascades of the mTOR signalling pathway and its downstream physiological outputs, enabling us to link dietary arginine with responses at the molecular, cellular, and phenotypic levels. We investigated how dietary arginine modulates life-history traits (body mass) and fitness traits (egg production) and hepatic gene expression in adult female quails. We focused on mTOR signalling pathway genes, which are interconnected but have distinct functions. The mTOR signalling complex senses amino acid availability to regulate cell growth and metabolism. Its downstream target, *EIF4EBP1*, represses translation under nutrient limitation but promotes protein synthesis when *MTOR* is active. For fatty acid metabolism, *FASN* drives *de novo* synthesis, while *FABP1* facilitates intracellular transport. Another representative candidate genes of the mTOR downstream pathways are autophagy genes *ATG5* and *ATG9A*, which support autophagosome formation during nutrient stress. The other downstream effector is antioxidant defence genes *SOD2* and *SOD1*, which scavenge reactive oxygen species, and the growth axis was explored through *IGF1* and *GHR*, key regulators of systemic growth. This panel of genes reveals arginine’s impact on growth, production, cellular maintenance, and stress resilience in adult birds.

Amino acids such as lysine have been shown to affect fitness traits in avian species, including broilers (Nasr & Kheiri, 2017). Dietary arginine has also been reported to influence life-history traits and fitness traits in quail, as well as mTOR activity in several species, particularly in pigs, sheep, and mainly in broiler chickens (Ali et al., 2025; Bauchart-Thevret et al., 2010; Cuca & Jensen, 1990; D’Amato & Humphrey, 2010; Fathima et al., 2024; Kong et al., 2012; Liu et al., 2023; Maurício et al., 2016; Wang et al., 2022). Previous reports indicate that arginine supplementation does not affect performance parameters in 7-day-old quail (Kheiri & Landy, 2020). In contrast, our previous research on growing quails showed that dietary arginine modulates both body mass and regulates the mTOR pathway, including the upregulation of growth-related genes (Gashew et al., unpublished). However, it remains unclear whether similar mechanisms operate in adult Japanese quail, which must balance self-maintenance with reproductive investment. The current study aimed to uncover how adult nutritional availability shapes the integration of molecular responses with life-history trade-offs in adult birds by manipulating arginine levels (low, control, high) and analysing gene expression. Thus, we hypothesised that dietary arginine levels modulate body mass, egg production, as well as hepatic expression of candidate genes representing major mTOR-regulated pathways in adult female quail. Specifically, we predicted that high arginine availability would upregulate growth-related (*MTOR, EIF4EBP1, IGF1, GHR*) and lipid metabolism genes (*FASN, FABP1*), while low arginine would increase the expression of autophagy (*ATG5, ATG9A*) and antioxidant defence genes (*SOD2, SOD1*).

## 2 Materials and methods

### 2.1 Animal management

Japanese quail chicks were hatched and reared at the Animal House of the Institute of Animal Science, Biotechnology, and Nature Conservation, University of Debrecen, Hungary (EU Directive 2010/63/EU). We hatched the birds in our farm using an industrial incubator (WQ-63 Model 2021 Version 2, AGROFORTEL, Budapest, Hungary) under standard conditions (Ndunguru et al., 2024b). Chicks had *ad libitum* access to feed and water until they reached six weeks of age. Infrared lamps maintained the initial rearing temperature of 37°C for four days. After that, it was lowered by 3°C every four days until it reached 24°C at the end of the six weeks. Relative humidity in the cage was from 60 to 65%. From the beginning of the experiment, the room was maintained at 24 ± 3°C, 60–75% relative humidity, and a 14:10 h light-dark cycle. The quails were fed a breeder diet formulated based on corn, wheat, corn germ meal, corn gluten meal, and soybean meal, containing 18% crude protein and 12.13 MJ/kg metabolizable energy (Table 1).

**Table 1:** Feed composition and calculated nutrient content of the experimental diets.

Ingredients, %	Treatments		
	Control	Low arginine	High arginine
Corn	9.53	9.53	9.53
Wheat	20.00	20.00	20.00
Corn germ meal	34.28	34.28	34.28
Corn gluten meal	2.24	2.24	2.24
Soybean meal	12.81	12.81	12.81
Sunflower oil	11.91	11.91	11.91
Limestone	5.93	5.93	5.93
MCP	1.01	1.01	1.01
L-Lys	0.38	0.38	0.38
DL-Met	0.15	0.15	0.15
L-Thr	0.11	0.11	0.11
L-Trp	0.02	0.02	0.02
L-Arg	0.39	0.00	0.78
Salt	0.35	0.35	0.35
Inert (Kaolin)	0.39	0.78	0.00
Premixture	0.50	0.50	0.50
Nutrient content (%)			
ME, MJ/kg	12.13	12.13	12.13
Crude protein	18.00	18.00	18.00
Lys	1.000	1.000	1.000
Met	0.450	0.450	0.450
Met+Cys	0.781	0.781	0.781
Thr	0.740	0.740	0.740
Trp	0.190	0.190	0.190
Leu	1.469	1.469	1.469
Ile	0.668	0.668	0.668
Arg	1.260	0.945	1.575
Leu/Ile	2.200	2.200	2.200
Ca	2.500	2.500	2.500
P	0.599	0.599	0.599
non phytate P	0.350	0.350	0.350
Na	0.150	0.150	0.150
DCAB, mEq/kg	96.7	96.7	96.7

aThe premix provided the following per kilogram of complete diet: 5000 IU vitamin A, 1000 IU vitamin D_3_, 24.5 mg/kg vitamin E, 1 mg vitamin K_3_, 0.75 mg vitamin B_1_, 2.5 mg vitamin B2, 6 mg Ca-d-Pantothetane, 2 mg vitamin B_6_, 10 ug vitamin B_12_, 55 µg biotin, 12.5 mg niacin, 0.3 mg folic acid, 1500 mg choline chloride, 66 mg Zn, 9.6 mg Cu, 48.1 mg Fe, 66 mg Mn, 0.9 mg I, 0.21 mg Se, 60 µg Co. MCP: monocalcium phosphate; ME: metabolisable energy; DCAB: dietary cation-anion balance (electrolyte balance); The isoleucine level of the control diet was similar to the recommendation of Santos et al. (2016), while the other amino acid levels was similar or exceeded the values set by the NRC, 1994.

### 2.2 Experimental design and sampling

At six weeks of age, 81 female Japanese quails, approximately similar in size (242.65 ± 6.7 g), were selected and randomly assigned to three treatment groups. The experiment followed a completely randomised design with three treatments and three replicates, each containing nine birds. The dietary treatments included a control group: a diet formulated per National Research Council (U.S.). Subcommittee on Poultry Nutrition (1994), a high arginine group (1.575%): control diet supplemented with 25% more arginine than the recommended amount, and a low arginine group (0.945%): control diet restricted by 25% of arginine from the recommended amount (Table 1). Quails were housed in groups with identical cages (45 cm × 52 cm × 27 cm; length × width × height) in the same room to ensure consistent environmental conditions. They were individually tagged with numbered plastic rings. The trial was conducted for 14 days, a duration previously shown to be sufficient to detect alterations in gene expression (Reda et al., 2024a). Live body mass was measured using a digital balance (± 0.1 g accuracy) on days 0 (initial), 7 (midpoint), and 14 (final) to assess body mass across treatments. Egg number and egg mass were recorded daily across the trial period.

On day 14 of the trial, 24 birds (eight per treatment) were euthanised through cervical dislocation for tissue sampling. Liver tissues were immediately collected, placed in sterile tubes, flash-frozen, transported to the laboratory, where they were stored at −80°C until analysis.

### 2.3 RNA extraction and purification

Total RNA was extracted from liver tissues using the peqGOLD Total RNA Kit (VWR, Radnor, PA, USA) with on-column DNase I digestion, following the manufacturer’s instructions. RNA concentration and purity were assessed using a Synergy HT Multi-mode Microplate Reader (BioTek Instruments, Winooski, VT, USA). Integrity was checked with a Qubit 4 fluorometer (Invitrogen™).

Complementary DNA (cDNA) was synthesised in a PCRmax AlphaThermal Cycler (Cole-Parmer Ltd., Vernon Hills, IL, USA) using the LunaScript® RT SuperMix Kit (New England Biolabs Inc., Ipswich, MA, USA) with 800 ng RNA, 5x LunaScript RT Supermix, and nuclease-free water in a 20 µL final volume. Thermal conditions included 25°C for 2 min (annealing), 55°C for 10 min (reverse transcription), and 95°C for 1 min (inactivation). cDNA was diluted 10-fold and stored at −20°C for qPCR.

Quail-specific, intron-spanning primers were designed using Oligo 7 software (version 7.6) and checked for target identity via the Primer BLAST web-based tool (NCBI, https://www.ncbi.nlm.nih.gov/tools/primer-blast/, accessed on January 28, 2025) (Table 2). qPCR was performed with AriaMx Real-Time PCR System (Agilent Technologies, Santa Clara, CA, USA) using HOT FIREPol® EvaGreen® qPCR Mix Plus (Solis BioDyne, Teaduspargi, Estonia) according to the manufacturer’s protocol. Each reaction included 5x HOT FIREPol® EvaGreen® qPCR Mix Plus (Solis BioDyne, Teaduspargi, Estonia), 8 ng cDNA template, 200 nM of each primer, and distilled water in a 10 µL final volume. Reactions were run in duplicates using 96-well plates (Sorenson 2633), with calibrators included to control for interplate variation, and no template controls were included for each primer. Cycling conditions were 95°C for 12 min, followed by 40 cycles of 95°C for 15 s, 60°C for 20 s, and 72°C for 20 s. Reference gene stability was assessed for *RPL19, GAPDH*, and *RN18S* using NormFinder, BestKeeper, and Delta Ct algorithms. *RPL19* was identified as the most stable and used for normalisation (Joshi et al., 2022; Simon et al., 2018). Relative expression of *MTOR, IGF1, GHR, FASN, FABP1, EIF4EBP1, ATG5* and *ATG9A*, and *SOD1* and *SOD2*was quantified as fold change using the 2^−ΔΔCt^ method (Livak & Schmittgen, 2001). Log-transformed expression values were used for statistical analysis.

**Table 2:** Primer design for the target and reference gene.

Gene	Gene name	Primer sequences (5´ = > 3´) Forward/reverse	Gene bank accession no.	Amplicon length range (bp)	Melting temperature (Tm)
*MTOR*	Mechanistic target of rapamycin	F: CCG AAG CAT TGA ATT GGC CCT R: CAT CTC TCA AAG GCA GCG GAC C	XM_015882433.2	116	F:61.57 R: 63.50
*IGF1*	Insulin-like growth factor 1	F: CAC TAT GCG GTG CTG AGC TGG TT R: ATC CCC TTG TGG TGT AAG CGT CT	XM_015867574.2	118	F: 65.42 R: 63.80
*GHR*	Growth hormone receptor	F: GGC ACT GGT CTG TGT GAA TGA CT R: CCA GCT CAG GTG ATC TGC ACT T	XM_032441512.1	89	F: 62.93 R: 62.58
*FASN*	Fatty acid synthase	F: TCA GCC CGA ACC TCC GCC AT R: ATG CCT GCA ATC ACC ACG TCT	XM_015879647.2	72	F: 66.24 R: 62.67
*FABP1*	Fatty acid synthesis binding protein 1	F: AGT CCC ATG AGA ACT TTG AGC CTT R: CTG GAT CTG TTC ATC AGG AAG CCC	XM_015862881.2	62	F: 62.04 R: 63.03
*EIF4EBP1*	Eukaryotic Translation Initiation Factor 4E-Binding Protein	F: ACC AGC CCA ATT GTG GAG GAG TT R: CTC AGG GCA CGT GCT TTA GAT GT	XM_015883175.1	120	F: 64.29 R: 63.03
*ATG5*	Autophagy-related gene 5	F: ATA GTG GAT TTC GGT ACA TCC CA R: TCC TCC AGA AGC AAT TGG TCG	XM_015858735.1	95	F: 59.03 R: 60.34
*ATG9A*	Autophagy-related gene 9	F: CAA CGC CCT CAG GAT CCC CAT R: ACG ATG CGG GCC TGT ACC TCC	XM_015868966.2	69	F:56.8 R: 59
*SOD2*	Superoxide dismutase 2	F: ACA GCA AAC ACC ACG CCA CCT R: AGC GAC ACC TGA GCT GTA ACA TC	XM_015858046.1	100	F: 65.38 R: 62.77
*SOD1*	Superoxide dismutase 1	F: CAA GCA GCA CGG TGG ACC AA R: TTC GCA GTC ACA TTG CCG AGG T	XM_015881247.1	66	F: 63.57 R:65
*RPL19*	Ribosomal protein L19	F: CAT CGG TAA GAG GAA GGG T R: ACG TTG CCC TTG ACC TTC AG	XM_015885843.1	162	F: 55.80 R: 60.54

### 2.4 Statistical analysis

All statistical analyses were performed using R v. 4.2.2 (R Core Team, 2024). Data visualisation was conducted using the ‘ggplot2’ package (version 3.4.3).

To evaluate the effects of treatment, time, and their interaction on body mass over the 14-day trial period, we fitted linear mixed models (LMMs) using the ‘lmer’ function from the ‘lme4’ package (Bates et al., 2015), where individual bird identity is treated as a random factor. For hen-day egg production and egg mass, we used a linear mixed model considering cage number as a random factor. HDEP (%) = (Number of eggs produced in a day / Number of live hens on that day) × 100 (Emam, 2021; Narinc et al., 2013). The best-fitting model was selected using Akaike’s Information Criterion corrected (AICc) (Vaida & Blanchard, 2005). The significance of fixed effects was assessed using the ‘lmerTest’ package (version 3.1.3) to compute p-values using two-way ANOVA (Kuznetsova et al., 2017). Post-hoc pairwise comparisons of means were performed using estimated marginal means via the ‘emmeans’ package with Tukey’s HSD test at *p* < 0.05 significance level (Kuznetsova et al., 2017; Searle et al., 1980). Linear models were employed to examine the effects of treatment on the expression of target genes.

Gene network connectivity describes the correlation structure between gene expression profiles (Langfelder & Horvath, 2008). In the analysis, degree centrality measures how many direct connections a gene has, emphasising the importance of connections with high-degree genes (hubs) to the overall network function. Betweenness centrality reflects a gene’s role as a bridge, indicating how often it lies on the shortest paths between other genes. In contrast, closeness centrality assesses how close a gene is to all other genes in the network, providing insight into the efficiency of information flow through that gene. In this network, genes are represented as nodes, and nodes are connected when there is a high level of co-expression between the corresponding genes (Zhang & Horvath, 2005) and using the ‘igraph’ package in R.

### 2.5 Ethical approval

The experiment was conducted in compliance with the EU Directive 2010/63/EU on the protection of animals used for scientific purposes. All procedures adhered to institutional guidelines and regulations. Approval was obtained from the Ethical Committee for Animal Use at the University of Debrecen, Hungary (Protocol No. 5/2021/DEMAB). The reporting of this study follows the ARRIVE (Animal Research: Reporting of In Vivo Experiments) guidelines.

## 3 Results

### 3.1 Effect of treatments on body mass

**Fig. 1:**
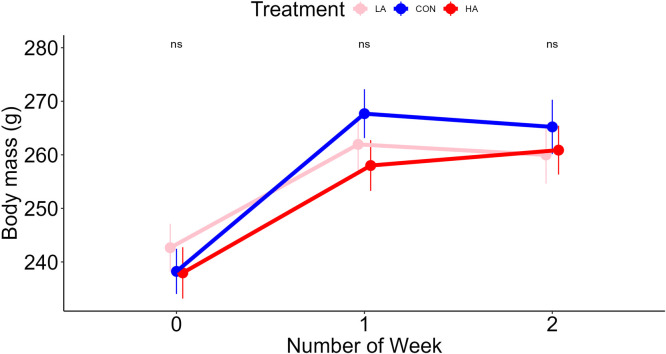
Effect of treatments across time on body mass. Data are means ± SEM, analysed using two-way ANOVA. n.s., not significant at p < 0.05. CON control, HA high arginine, LA low arginine. Number of weeks: 0: week 0, 1: week 1, 2: week 2.

Body mass increased in all groups during the first week and then remained unchanged in the second week, with no difference between the treatment groups at any time point (Fig. 1; Table 3).

**Table 3:** Analysis of variance for the effect of treatments and time on body mass.

	Sum Sq	Mean Sq	NumDF	DenDF	F-value	*p*-value
Treatment	131.3	65.7	2	78	0.321	0.725
Time point	27765.6	13882.8	2	156	68.029	<0.001
Treatment: time point	1154.3	288.6	4	156	1.4141	0.2318

### 3.2 Egg number and egg mass

**Fig. 2:**
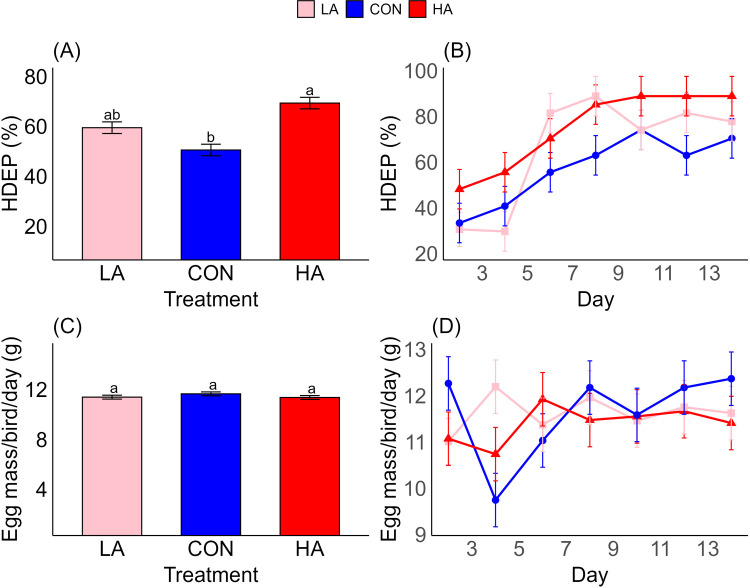
Effect of treatment and day on egg number and mass. (A) effect of treatment on hen-day egg production (HDEP), (B) effect of day on HDEP, (C) effect of treatments on egg mass/bird/day, (D) effect of day on egg mass/bird/day. Data are means ± SEM. Different letters indicate significant differences at a given time point at *p* < 0.05. LA: low arginine, CON: control, HA: high arginine.

Hen-day egg production continued to increase in the arginine-supplemented groups throughout the trial period (Fig. 2A, B; Table 4), whereas egg mass remained unchanged across all treatments during the same period (Fig. 2C, D; Table 4).

**Table 4:** Analysis of variance for the effect of treatments and time on hen-day egg production and egg mass.

Effects	Df	DenDF	F-value	*P*-value
HDEP				
Treatment	2	6.0	7.47	0.023
Day	13	78.1	15.72	<0.001
Treatment: day	26	78.1	1.23	0.238
Mass/bird/day				
Treatment	2	6.0	0.23	0.794
Day	13	78.0	1.63	0.094
Treatment: day	26	78.0	1.25	0.222

### 3.3 Effect of dietary arginine on the mTOR signalling pathway gene expression

**Fig. 3:**
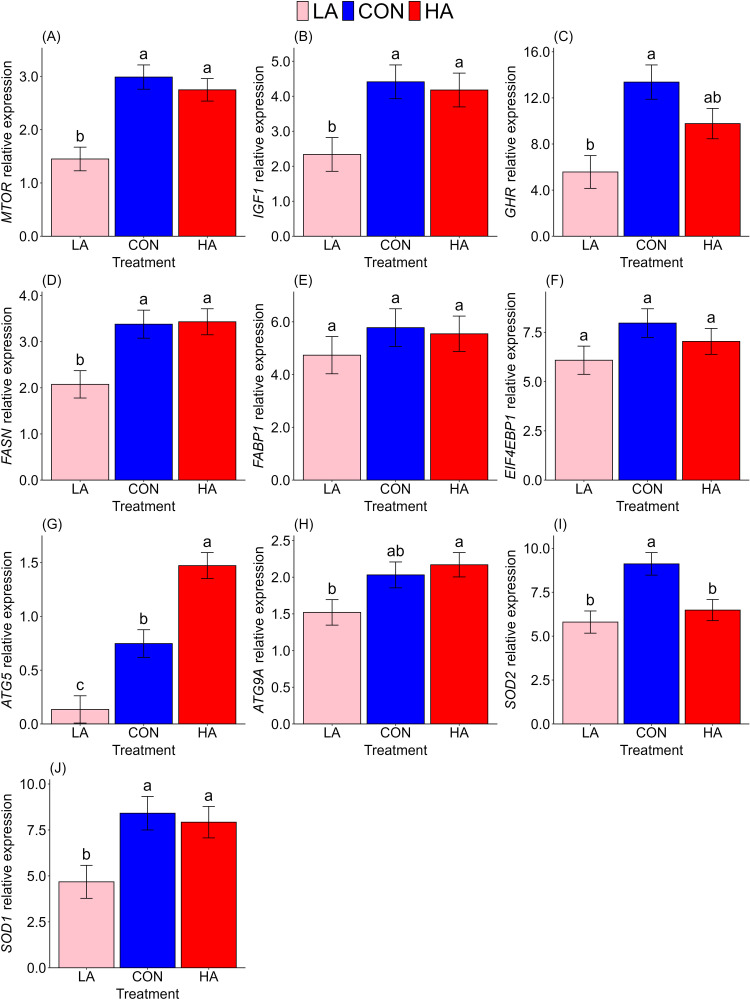
Effect of dietary arginine on gene expression. (A) *MTOR*, mechanistic target of rapamycin. (B) *IGF1*, insulin-like growth factor 1; (C) *GHR*, growth hormone receptor; (D) *FASN*, fatty acid synthase; (E) *FABP1*, fatty acid synthesis binding protein 1; (F) *EIF4EBP1*, eukaryotic translation initiation factor 4e-binding protein 1; (G) *ATG5*, autophagy-related 5 gene; (H) *ATG9A*, autophagy-related 9 gene (I) *SOD2*, superoxide dismutase 2; (J) *SOD1*, superoxide dismutase 1. Data are means ± s.e.m. from 8 birds per group were analysed using ANOVA and a linear model. Different letters indicate significant differences at a given time point at *p* < 0.05. LA low arginine, CON control, HA high arginine. Note the different scales among panels.

Dietary arginine significantly affected the expression of genes involved in the mTOR signalling pathway in adult Japanese quail (Fig. 3). Except for *FASBP*1 and *EIF4EBP1*, arginine restriction resulted in downregulation of all genes related to growth, autophagy, and antioxidant responses, including *MTOR* (*p* < 0.001), *IGF1* (*p*= 0.006), *GHR* (*p*= 0.003), *FASN* (*p*= 0.009), *ATG5* (*p*= 0.004), *ATG9A* (*p*= 0.063), *SOD2* (*p*= 0.002), and *SOD1p*= 0.011). Conversely, arginine supplementation led to an upregulation of *ATG5* (*p* < 0.001) and a downregulation of the antioxidant gene *SOD2* (*p*= 0.007).

### 3.4 Gene networking analysis

**Fig. 4:**
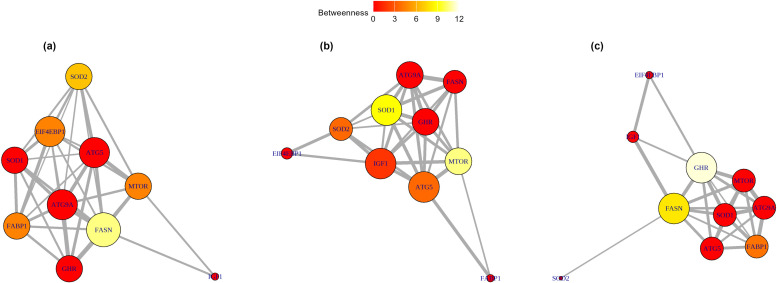
Gene network analysis. (a) low arginine treatment, (b) control treatment, (c) high arginine treatment. *MTOR*, mechanistic target of rapamycin; *IGF1*, insulin like growth factor 1; *GHR*, growth hormone receptor; *FASN*, fatty acid synthase; *FABP1*, fatty acid synthesis binding protein 1; *EIF4EBP1*, Eukaryotic Translation Initiation Factor 4E-Binding Protein 1; *ATG5*, autophagy related gene 5 gene; *ATG9A*, autophagy-related gene 9 gene, *SOD1 & SOD2*, superoxide dismutase 1, 2, respectively. Each gene is represented as a node, and correlations between gene expression values (r > 0.3) are represented as edges. The node size (ranges from 0–12, where 0 degree is no connectedness, whereas 12 degree is high connectedness) reflects the degree of centrality, which indicates how many direct connections a gene has, emphasising the importance of connections with high-degree genes (hubs) to the overall network function. Betweenness centrality is represented by node colour, with cream indicating high and red indicating low centrality, respectively. Betweenness centrality reflects how often a gene lies on the shortest paths between other genes. Larger, cream coloured node represent central gene with many connections and high bridging potential. The edge thickness corresponds to the strength of the correlation between genes.

Gene co-expression topology differed markedly among treatments (Fig. 4, Table 5), indicating that arginine availability reshapes how mTOR-related genes co-vary with one another. Under arginine restriction (Fig. 4a), co-expression was dominated by lipid metabolism and translation-related genes. *FASN* had the highest degree (9) and the largest betweenness (5), marking it as the dominant hub and an important bridge in the arginine-restricted network. Oxidative stress gene *SOD2* and *EIF4EBP1* showed the greatest closeness values (0.220 and 0.213, respectively), indicating they are relatively proximal to other genes in the low-arginine network. Several autophagy genes (*ATG5, ATG9A*) and *MTOR* remained well connected (degree 7–8), but the topology under restriction emphasises *FASN* and *EIF4EBP1* as prominent central players and *SOD2* as a close, bridging node. In the control group (Fig. 4b), the network was more balanced compared to arginine restriction, with multiple genes showing a high degree (*IGF1, ATG5* and *SOD1*, all= 8). *MTOR* displayed a notable betweenness (6), consistent with a mediating role under adequate nutrition. Under arginine supplementation, *GHR* and *FASN* emerged as central nodes, each with a high degree and strong closeness, while *GHR* also exhibited particularly strong betweenness (12), identifying it as a key bridging node in the supplemented network. In contrast, stress-responsive genes such as *SOD2* lost centrality under supplementation, indicating a reduced role for this stress-related gene under supplementation.

**Table 5:** The effect of treatments on centrality measures.

Gene	Degree			Betweenness			Closeness		
	LA	CON	HA	LA	CON	HA	LA	CON	HA
*MTOR*	7	7	6	2	6	0	0.179	0.160	0.132
*IGF1*	2	8	3	0	1	0	0.134	0.172	0.124
*GHR*	7	7	8	0	0	12	0.165	0.155	0.177
*FASN*	9	6	8	5	0	8	0.202	0.148	0.177
*FABP1*	7	2	6	2	0	4	0.191	0.117	0.176
*EIF4EBP1*	8	3	2	2	0	0	0.213	0.127	0.111
*ATG5*	8	8	6	0	2	0	0.173	0.161	0.159
*ATG9A*	8	7	6	0	0	0	0.183	0.144	0.145
*SOD1*	7	8	6	0	5	0	0.181	0.175	0.133
*SOD2*	7	6	1	3	2	0	0.220	0.156	0.124

LA low arginine, CON control, HA high arginine.

## 4 Discussion

In the present study, dietary arginine supplementation led to an increase in egg production (Fig. 2A). However, neither arginine restriction nor supplementation led to a significant effect in body mass or egg mass (Figs. 1; 2C, respectively). Despite this lack of observable phenotypic variation, significant alterations in the mTOR pathway gene expression indicate that dietary arginine may influence molecular processes independently of physiological outcomes. Although body mass and egg mass were unaffected in adults, birds’ dependency on dietary arginine is still critical because they lack a complete urea cycle and cannot synthesise arginine efficiently (Fernandes & Murakami, 2010; Silva et al., 2012). This metabolic limitation likely explains the pronounced molecular and metabolic effects of arginine restriction even in the absence of measurable changes in body or egg mass.

These findings align with previous studies showing variable effects of dietary arginine on avian reproduction. For instance, optimal intake levels of arginine are associated with improved egg production (de Lima et al., 2022; Sousa et al., 2022), while higher concentrations (up to 1.755%) showed no improvement in egg mass (Maurício et al., 2016; Morais et al., 2022; Tuesta et al., 2018). Arginine's effect on growth and gene expression varies by dietary amount and tissue type (Ghiasvand et al., 2025).

The mTOR signalling pathway, a key nutrient-sensing system (Laplante & Sabatini, 2012; Regan et al., 2020), is modulated by amino acids like leucine, methionine (Ndunguru et al., 2024a; Ndunguru et al., 2024b), and arginine (Jung et al., 2019; Wang et al., 2015). Dietary restriction, similar to other nutritional shifts, adjusts mTOR activity, triggering adaptive changes in hepatic gene expression to maintain metabolic balance (Feige-Diller et al., 2022; Reda et al., 2024a). Previous findings showed that arginine restriction in juvenile Japanese quail reduced body mass in both sexes and upregulated *MTOR* expression in males (Gashew et al., unpublished). In contrast, currently, adult female quail demonstrated significantly reduced *MTOR* gene expression under restriction (Fig. 3A), highlighting mTOR's role in nutrient sensing and metabolic regulation. Unlike juveniles, which prioritise rapid somatic growth, adults allocate nutrients toward reproduction and self-maintenance, making body mass less sensitive to arginine levels. Thus, mTOR signalling changes in adults are primarily molecular, supporting reproductive performance as previously mentioned.

The mTOR signalling pathway is intricately linked to the GH-IGF1 axis, a conserved endocrine pathway that regulates somatic growth and tissue differentiation across vertebrates (Fu et al., 2001). In birds, hepatic IGF-1 production is stimulated by GH and modulated by nutritional status, with elevated *IGF1* mRNA levels observed in the quail oviduct during sexual maturation, indicating a dual role in growth and reproduction (Kida et al., 1994). Our findings support this report: arginine restriction significantly suppressed *MTOR* expression and concurrently reduced *IGF1* (Fig. 3B) and *GHR* (Fig. 3C) gene expression, suggesting that arginine availability directly influences the GH-IGF-1-mTOR axis. This molecular regulation likely enhances metabolic efficiency and supports the observed reproductive performance, suggesting that arginine, via the mTOR-GH-IGF1 axis, prioritises nutrient allocation to reproduction over somatic maintenance in adults.

In avian species, intracellular lipid-binding proteins are notably abundant in the liver, regulate lipid metabolism (Murai et al., 2009; Na et al., 2018). Fatty acids are essential for fat synthesis, and the *FABP* gene encodes intracellular lipid-binding proteins that support energy homeostasis and tissue function (Donnelly et al., 2005; Mao et al., 2020; Storch & Corsico, 2008; Wang et al., 2019). Lipid is especially important in birds, where the efficient mobilisation and utilisation of fatty acids are essential for yolk formation and sustaining reproductive output (Blem, 1976; Ramenofsky, 1990; Ruan et al., 2015). In adult female Japanese quail, we observed that arginine restriction led to a downregulated *FASN* gene expression (Fig. 3D), indicating reduced de novo lipid synthesis, while *FABP1* expression remained unchanged (Fig. 3E). This suggests that arginine availability regulates lipid synthesis but not transport, likely due to stable baseline needs or compensatory mechanisms. As previously reported, arginine restriction suppressed *MTO*R and *IGF1* expression, which aligns with reduced FASN activity, indicating a shift from lipid accumulation to energy conservation. This molecular response supports the prioritisation of the evidenced reproductive output, underscoring arginine’s role in modulating lipid metabolism under nutritional stress.

Autophagy, a crucial cellular recycling mechanism, plays a significant role in the stress response and feed efficiency of avian species, enabling them to adapt to nutritional challenges. Research has shown that autophagy-related genes are expressed across various tissues in chickens and quails, with distinct variations influenced by gender and genotype (Piekarski et al., 2014). Nutrient restriction modulates antioxidant enzyme activity and gene expression, maintaining cellular homeostasis (Wu et al., 2020). These adaptive mechanisms underscore autophagy’s essential function in maintaining cellular homeostasis under fluctuating dietary conditions. In our study, arginine restriction resulted in the downregulation of *ATG5* (Fig. 3G) and *ATG9A* (Fig. 3H) genes, vital for autophagosome formation and maintenance, suggesting that reduced arginine availability hinders the autophagy process and may limit the quail’s capacity to recycle cellular components during stress. Simultaneously, antioxidant gene expression was affected, with arginine restriction downregulating *SOD1* expression (Fig. 3J), while both restriction and supplementation led to downregulated *SOD2* (Fig. 3I) expression. These findings align with prior suppression of *MTOR, IGF1*, and *GHR* expression and *FASN* downregulation, indicating a metabolic shift from growth and lipid synthesis to energy conservation. Although mTOR inhibition typically promotes autophagy, reduced *ATG5* and *ATG9A* expression suggest that arginine restriction limits autophagic recycling, likely prioritising energy allocation to reproductive output. Similar *SOD2* expression under both low and high arginine levels suggests a homeostatic adjustment in oxidative metabolism. This indicates that arginine deficiency or excess modulates mitochondrial function, leading to a biphasic antioxidant response rather than a linear dose-dependent pattern (Kalvandi et al., 2022).

Gene expression analysis is widely used to explore the molecular basis of phenotypic traits, but examining differential expression alone can be limiting, as it does not account for how genes interact within broader networks. Moreover, many genes contribute to multiple pathways, phenotypes, or subnetworks (Frings et al., 2012), and no single experimental approach can fully capture all connections within the interactome (Alexeyenko & Sonnhammer, 2009). The gene network analysis (Fig. 4, Table 5) provided insight into how dietary arginine reshaped the organisation of genes within the MTOR signalling pathway. Under arginine restriction, several growth-related genes (*MTOR, IGF1, GHR, FASN*) and autophagy genes (*ATG5, ATG9A*) were downregulated, along with the antioxidant genes *SOD1* and *SOD2*. Despite reduced expression, *SOD2* retained a key bridging role, underscoring the continuing importance of antioxidant pathways in maintaining cellular homeostasis under nutrient stress. *FASN* and *EIF4EBP1* emerged as central nodes, reflecting their role in sustaining lipid metabolism and translational control even when growth and autophagy were suppressed. In the control diet, where growth- and antioxidant-related genes exhibited moderate expression, *IGF1* and *SOD1* occupied central positions, indicating a balanced integration of growth and oxidative stress regulation under adequate nutrition. By contrast, with arginine supplementation, the upregulation of *ATG5* and downregulation of *SOD2* coincided with a shift in network centrality toward *GHR* and *FASN*, emphasising growth-promoting and lipogenic processes while reducing reliance on oxidative stress defences. These reorganisations were consistent with the observed increase in HDEP. We interpret these topology changes as descriptive evidence that arginine availability alters the coordination between growth, lipid metabolism, autophagy and oxidative-stress genes, rather than as proof of directional regulatory control. Overall, arginine supplementation appeared to shift resource allocation toward reproductive performance rather than somatic maintenance, underscoring its complex regulatory role in avian physiology and the need for further study under varying nutritional conditions.

## 5 Conclusion

Our study demonstrated that dietary arginine had distinct effects on adult Japanese quail depending on whether it was restricted or supplemented. While arginine supplementation increased hen-day egg production, neither supplementation nor restriction significantly altered body mass or egg mass, indicating that reproductive investment may be enhanced independently of somatic maintenance. At the molecular level, arginine restriction led to a downregulation of genes in the mTOR–GH–IGF-1 axis, as well as those involved in lipid synthesis (*FASN*), autophagy (*ATG5, ATG9A*), and antioxidant defence (*SOD1, SOD2*), pointing to a coordinated metabolic suppression under nutrient stress. In contrast, supplementation elevating *ATG5* and shifted gene network centrality toward growth and lipid-related genes (*GHR, FASN*), aligning with the observed increase in hen-day production percentage. Overall, these results indicated that arginine availability influences how metabolic resources are allocated in adult birds. Restriction suppresses both anabolic and catabolic processes, whereas supplementation enhances reproductive output through specific molecular adjustments. These findings highlight the need to evaluate nutritional effects beyond conventional growth measures to better understand the molecular trade-offs that shape reproductive performance.

## Data availability statement

The raw data supporting the conclusions of this article will be made available by the authors, without undue reservation.

## Funding

The study was supported by the National Development, Research and Innovation Office, Hungary (K139021). M.G., F.N.A., and E.M.A.. are supported by a Stipendium Hungaricum Scholarship from Tempus Public Foundation for PhD studies.

## Ethical approval

The experiment was conducted in compliance with the EU Directive 2010/63/EU on the protection of animals used for scientific purposes. All procedures adhered to institutional guidelines and regulations. Approval was obtained from the Ethical Committee for Animal Use at the University of Debrecen, Hungary (Protocol No. 5/2021/DEMAB). The reporting of this study follows the ARRIVE (Animal Research: Reporting of In Vivo Experiments) guidelines.

## CRediT authorship contribution statement

**Mequanint Gashew:** Writing – original draft, Visualization, Methodology, Formal analysis, Data curation, Conceptualization. **Gebrehaweria K. Reda:** Writing – review & editing, Visualization, Formal analysis. **Fadella Nur Almira:** Writing – review & editing. **Eman Moustafa Abdelbary:** Writing – review & editing. **Gabriella Gulyás:** Writing – review & editing. **Renáta Knop:** Writing – review & editing. **Brigitta Csernus:** Writing – review & editing. **Csaba Szabó:** Writing – review & editing, Methodology. **Ádám Z. Lendvai:** Writing – review & editing, Visualization, Supervision, Project administration, Methodology, Investigation, Data curation, Conceptualization. **Levente Czeglédi:** Writing – review & editing, Supervision, Project administration, Investigation, Data curation, Conceptualization.

## Declaration of competing interest

The authors declare that they have no known competing financial interests or personal relationships that could have appeared to influence the work reported in this paper.
